# Rapid production of the anaesthetic mepivacaine through continuous, portable technology†

**DOI:** 10.1039/d3gc04375d

**Published:** 2024-01-23

**Authors:** Pablo Díaz-Kruik, Francesca Paradisi

**Affiliations:** a Department of Chemistry, Biochemistry and Pharmacology, University of Bern Freistrasse 3 Bern Switzerland francesca.paradisi@unibe.ch

## Abstract

Local anaesthetics such as mepivacaine are key molecules in the medical sector, so ensuring their supply chain is crucial for every health care system. Rapid production of mepivacaine from readily available commercial reagents and (non-dry) solvents under safe conditions using portable, continuous apparatus could make an impactful difference in underdeveloped countries. In this work, we report a continuous platform for synthesising mepivacaine, one of the most widely used anaesthetics for minor surgeries. With a focus on sustainability, reaction efficiency and seamless implementation, this platform afforded the drug in 44% isolated yield following a concomitant distillation–crystallisation on a gram scale after *N*-functionalisation and amide coupling, with full recovery of the solvents and excess reagents. The use of flow chemistry as an enabling tool allowed the use of “forbidden” chemistry which is typically challenging for preparative and large scale reactions in batch mode. Overall, this continuous platform presents a promising and sustainable approach that has the potential to meet the demands of the healthcare industry.

## Abstract 2

Local anaesthetics such as mepivacaine are key molecules in the medical sector, so ensuring their supply chain is crucial for every health care system.

## Graphical abstract

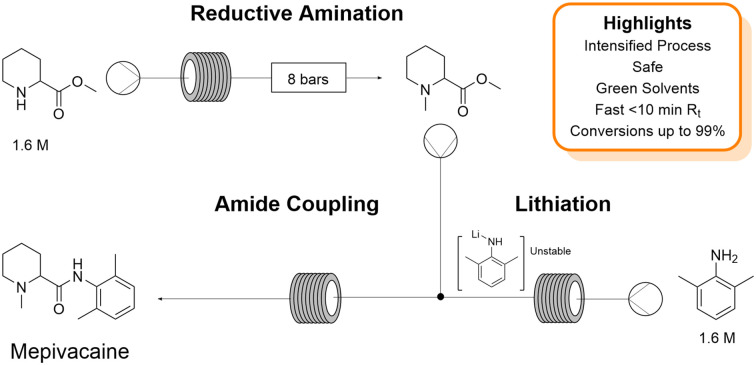

## Introduction

Local anaesthetics are a class of molecules that are regularly used for medical and dental procedures due to their ability to block nerve impulses and avoid the transmission of pain signals to the brain. Numerous academics and industries have shown significant interest in these molecules, and several synthetic methods are available.^1–3^

Mepivacaine and its derivatives such as ropivacaine and bupivacaine are among the most used agents in orthopaedic regional anaesthesia for lower limb surgeries as well as in oral and maxillofacial surgeries.^4–6^ Scarily, this is not the case in underdeveloped countries, where the use of local anaesthetics is not the standard practice for a variety of reasons such as the lack of medical training, availability of medications, storage, *etc*.^7,8^ The COVID-19 pandemic in 2020 has also highlighted in wealthy countries the critical need to establish autonomous chemical production of pharmaceuticals and chemicals to avoid supply chain shortages. Anaesthetics and sedatives, which are generally simple chemical structures, became extremely scarce in the pandemic,^9,10^ with the unprecedentedly high demand worldwide further aggravated by the disruption in production (and export) in India and China where a large percentage of raw ingredients are produced.^11–13^

To achieve rapid relocalisation of production, flow chemistry emerges as a key technology in this transition. Its intrinsic modularity and fast implementation set it apart from classical batch synthesis that usually requires large facilities.

**Fig. 1:**
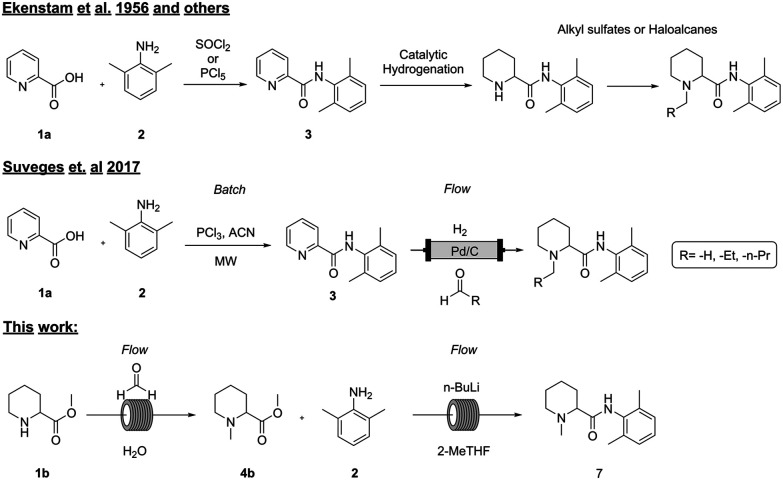
Comparison between the existing synthetic methods and our work.

Several methods (Fig. 1) for the synthesis of these types of molecules have been described in the literature, first by Ekenstam *et al.* in 1956 and sixty years later by Suveges *et al.* in 2017.^1,14^ Generally, the first step is an amide coupling reaction between the aromatic picolinic acid 1a and 2 either *via* an acyl chloride intermediate or by using coupling reagents such as EDC/HOBt and DCC,^15,16^ and the second step is usually a palladium mediated hydrogenation to reduce the aromatic compound, followed by the alkylation of the pyrimidine nitrogen with a suitable alkylating reagent. Even though this strategy is efficient (85–95% crude isolated yields), it poses obvious environmental and operational risks on higher scales due to the handling of toxic reagents such as thionyl chloride, phosphorus pentachloride, and alkylating reagents that are well known to be potentially carcinogenic.^17,18^ In addition, from a process chemistry perspective the synthesis itself has several challenges: formation of the acyl chloride is an exothermic reaction that generates HCl_(g)_ and needs to be carefully controlled; otherwise, the risk of a runaway scenario could have fatal consequences.

In 2017, Suveges *et al.*^14^ redesigned the synthesis of these analogues, overcoming some of the risks and limitations of the previous strategies. In this case, the authors managed to spare one synthetic step by performing at the same time the hydrogenation and the *N*-alkylation *via* a reductive amination strategy, avoiding the use of toxic alkylating reagents. By taking advantage of a flow setup, they successfully used the same heterogeneous catalyst (10% Pd/C) and reducing agent (H_2(g)_) for both reactions, overcoming reagent toxicity and improving process safety. Nevertheless, this strategy still has some drawbacks such as the requirement of hydrogen (or hydrogen generators *in situ* such as an H-cube), generation of HCl_(g)_ upon amide coupling and the need to use PCl_3_ as a chlorinating reagent, leading to the formation of large amounts of phosphorus-derived waste that is difficult to process,^19^ lowering in this way the atom economy and greenness of the overall synthesis. In addition, the transposition to flow of the first step was not attempted due to the formation of precipitates that could lead to reactor clogging.

Here we present a continuous system which enables for the first time the rapid synthesis of these molecules which can be run in a completely automated manner, showcasing a safer and more sustainable alternative to the previously reported methods.

## Results and discussion

**Scheme 1:**
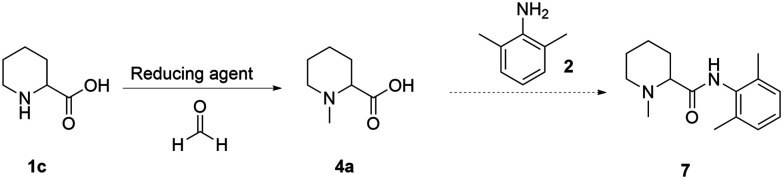
Overall reaction scheme for the synthesis of mepivacaine (7).

To date, all the reported strategies involve first the amide formation and then the reductive amination step (Fig. 1). In fact, the starting material is the aromatic picolinic acid, which upon reduction yields the saturated and racemic pipecolic portion of the final molecule. We have previously reported an efficient green continuous synthesis to obtain optically pure pipecolic acid starting from natural l-lysine, which affords the final product in excellent yields.^20^ In this work, starting from pipecolic acid (1c), we planned to swap the order of the reactions to avoid homocoupling between the free amine of the pipecolinate moiety and the carbonyl group, under our selected reaction conditions. Thus, performing the methylation first and the amide coupling second will minimise side product formation and increase the final product quality. Our initial strategy was designed as follows: starting from the free acid, performing the reductive amination, and carrying out the amide coupling under milder reaction conditions (Scheme 1).

### Reductive amination

Initial tests were carried out to understand if the reductive amination reaction had the potential to be transposed to flow. For this reason, different reducing agents were tested in batch mode (NaBH_3_CN, 2-picoline-borane, and formic acid) (Table 1).

**Table 1:** Batch mode optimal reaction conditions with different reducing agents for the *N*-functionalisation of pipecolinamine. Conversions were quantified by ^1^H-NMR

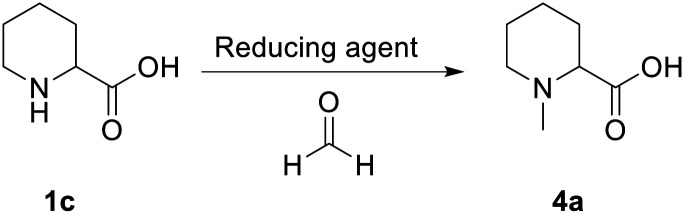				
Entry	Reducing agent (eq.)	Temperature (°C)	Reaction time (h)	Conversion (%)
1	NaBH_3_CN (1.5)	25	16	>99
2	2-Pic-borane (3.4)	25	0.08	>99
3	Formic acid (2650)	100	1	>99

**Scheme 2:**
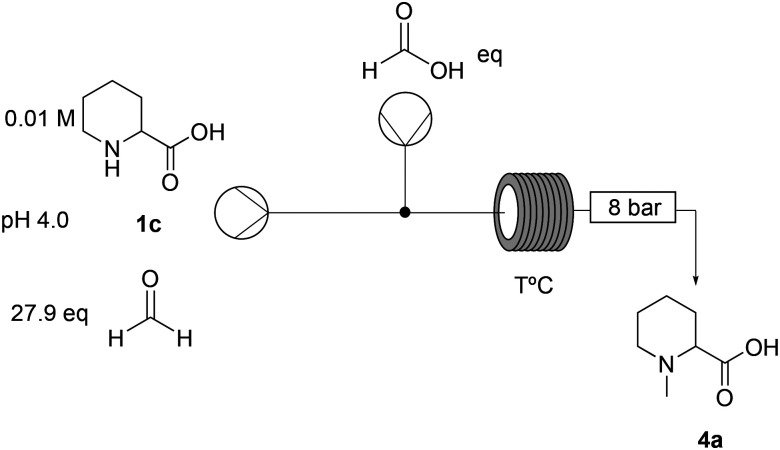
Flow setup for the *N*-functionalisation of pipecolic acid (1c).

All reagents, under the tested conditions, successfully reduced the imine bond. However, NaBH_3_CN, despite being efficient, displays health and process risks on large scales since toxic HCN_(g)_ could be released upon acidic workup; 2-picoline-borane is insoluble under the tested reaction conditions leading to potential clogging in a flow setup; finally the Eschweiler–Clarke^21^ reaction, which uses formic acid as a reducing agent, was found to be inefficient in terms of formic acid equivalents. Despite these limitations, the Eschweiler–Clarke approach has potential for a flow chemistry setup and it has been previously successfully applied to the continuous synthesis of dumetorine.^22^ Continuous flow apparatuses offer the possibility of working at temperatures higher than the boiling point of the solvent by controlling the pressure with a back-pressure regulator. Additionally, as all the reaction components and products are miscible, the risk of reactor fouling due to solid accumulation would be minimised. The first flow setup was therefore trialled (Scheme 2).

The first screening of the reaction conditions was performed in order to identify critical parameters (Tables S1–S4, ESI†). Residence time, formic acid equivalents, and reactor temperature were found to be crucial to achieve full conversion (entries 3 and 5, Table 2).

**Table 2:** Flow setup and reaction condition screening for the *N*-functionalisation. Reaction conditions: pipecolic acid (1c) (10 mM) solution was adjusted with 15% (v/v) acetic acid to pH 4, formic acid solution in H_2_O, formaldehyde 27.9 eq., 8 bar. Conversions were quantified by ^1^H-NMR

Entry	Molar ratio (pipecolic acid : formic acid)	Residence time (min)	Temperature (°C)	Conversion (%)
1	1 : 55	45	120	4
2	1 : 2650 (neat)	45	120	33
3	1 : 2650 (neat)	45	150	>99
4	1 : 2650 (neat)	15	150	80
5	1 : 1325 (50% in H_2_O)	45	150	>99

Once it was established that the system could efficiently perform under continuous flow, the second step of the multi-step synthesis, the amide coupling, was explored.

### Amide coupling

Generally, amide bond formation strategies require the activation of the carbonyl group to promote the attack of the amine. There are multiple activation strategies;^23^ the most common ones require the use of coupling reagents such as DCC, EDC, and HOBT, among many others. Other strategies involve the chlorination of the carboxylic acid to produce an acyl chloride, which is indeed the method of choice of the previously reported strategies due to its high efficiency.^1,14^ Once the intermediates are formed, they are typically reacted with the desired amine to afford the amide product. Even though these strategies are generally high yielding, they suffer from evident drawbacks regarding the environmental impact. For instance, they often involve the use of dimethylformamide (DMF) or dichloromethane (DCM), require high temperatures and generate large amounts of waste due to the need for coupling reagents. Additionally, workups and purification are usually challenging and the implementation on larger scales is therefore difficult.

Recent papers describe more sustainable amide bond syntheses; for example, in the work by Brittain *et al.* in 2021,^24^ they generated *in situ* an acyl fluoride to activate the carbonyl group. Pentafluoropyridine (PFP) is more efficient in terms of atom economy and solvent choice (acetonitrile) than the classically used coupling reagents such as HATU or PyBOP. However, with our substrate (1c), this approach was unsuccessful even with less hindered amines (Table S5, ESI†).

We revised our initial strategy and considered the possibility of starting from the commercially available methyl pipecolinate (1b) rather than the free acid, as this offers more options in terms of amide coupling. We seamlessly adapted the reductive amination protocol to the ester substrate (Table S7, ESI†) and tested the efficiency of an acyl transferase we had successfully used on other sterically hindered systems.^25,26^ Disappointingly, this did not lead to any detectable product (Table S6, ESI†).

**Scheme 3:**
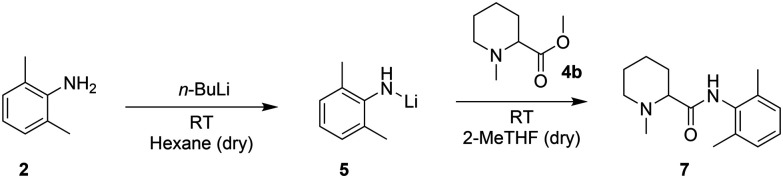
Hevia's protocol for the synthesis of amide bonds.

The Hevia group^27^ reported a fast and more sustainable strategy to produce amides starting from esters and *in situ* production of lithium amides in hexane (Scheme 3). They then replaced the widely used tetrahydrofuran (THF) with 2-methyltetrahydrofuran (2-MeTHF), a bio-derived solvent, for the coupling step.^28,29^ However, the requirements of pyrophoric reagents, cryogenic temperatures to control the exothermicity of the reaction, and dry and degassed solvents have so far limited the scalability of this system in batch. A continuous flow setup, with its highly efficient heat transfer and smaller reactor volumes, could offer a practical solution and could be tested with *N*-methylpipecolinate (4b).^30–33^

With our system, the use of hexane for the lithiation step (entries 1 and 2, Table 3) enabled such a reaction to be carried out at room temperature; however, the required solvent exchange from hexane to 2-MeTHF to carry out the coupling of the Li-amide with 4b appeared problematic for its transposition to flow. In batch, this is a straightforward task that only requires evaporation and subsequent solvent addition. In continuous flow, this is not possible; therefore, we tested the feasibility of the 2-step reaction in batch in a single solvent. For purely practical reasons, we screened different reaction conditions with available dry THF, with the intention of switching to the greener 2-MeTHF if the results were encouraging (2-MeTHF behaves virtually identical to THF in organometallic reactions requiring a strong Lewis base).^29^ The crude *N*-methylpipecolinate 4b (obtained in the previous step) could be easily redissolved in THF, but the use of THF as the sole solvent at room temperature for both lithiation and amide coupling was unsuccessful (entry 3, Table 3), leading to the deprotonation of THF and subsequent ring opening^34^ with significant heat generation. Lowering the temperature to −78 °C (entry 4, Table 3) showed no conversion after 2 h probably due to the low solubility of the reagents. In contrast, carrying out the deprotonation step at −78 °C and then allowing the reaction mixture to reach room temperature before amide coupling enabled the detection of the coupling product by ^1^H-NMR (entry 5, Table 3). Despite the poor efficiency of the reaction under the tested conditions and the requirement of a low temperature, the solubility was no longer an issue, and the reaction could be attempted in a flow setup.

**Table 3:** Batch reaction condition screening for the amide bond formation step. Deprotonation conditions: 1.0 eq. of *n*-BuLi from a 2.6 M solution, 2,6-dimethylaniline (2) 1.0 eq., 1 h. Amide coupling conditions: *N*-methylpipecolinate (4b) 1.0 eq. from a 0.8 M solution in THF. All reactions were performed under an inert atmosphere using dry and degassed solvents. Conversions were quantified by ^1^H-NMR

Entry	Solvent used for deprotonation	Temperature for deprotonation (°C)	Li-amide (1 M) (eq.) in THF	Temperature (°C) for coupling reaction	Conversion (%), time
1	Hexane	25	1.5	25	>99, 20 s a
2	Hexane	25	1.0	25	>99, 20 s a
3	THF	25	1.0	25	n.d., 2 h
4	THF	−78	1.0	−78	n.d., 2 h
5	THF	−78	1.0	25	Traces after 18 h

a 2-MeTHF was used in the coupling step.

To understand the behaviour of the reaction in flow, the system was decoupled into its two steps: Li-amide formation and amide coupling. To analyse the efficiency of the deprotonation step, this was carried out in flow followed by electrophilic quenching in batch using ethyl benzoate (4c) as a model substrate (Table S10 and Scheme S4, ESI†). To our surprise, this afforded the coupling product with excellent conversion at room temperature and with dry THF as the sole solvent within seconds. In flow, the heat exchange is highly favoured due to the decreased ratio between effective volume and heat exchange surface, leading to precise control of the reaction exothermicity.

**Fig. 2:**
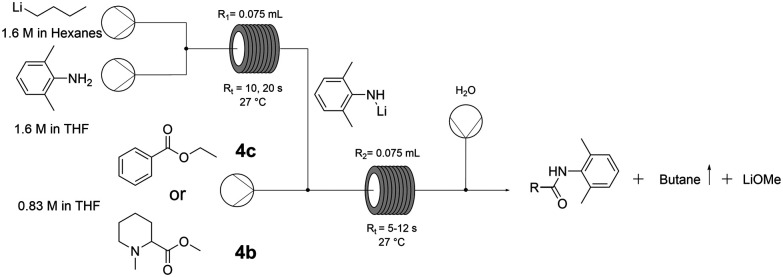
Flow setup for the amide coupling reaction.

We therefore telescoped both steps in continuous mode (Fig. 2), using first ethyl benzoate (4c) to optimise the conditions. 1.5 equivalents of Li-amide (5) to ester were found to be optimal, requiring just 10 seconds for the deprotonation step (entry 2, Table 4). The efficiency of mixing (higher conversion at shorter residence times, entries 2 and 3) is a crucial parameter in this very rapid process. The reaction was then tested with the crude pre-anaesthetic ester intermediate (4b) redissolved in dry THF. With this less activated substrate, increasing the residence time (*R*_t_) from 6 to 12 seconds for the amide coupling step was sufficient to achieve full conversion (entries 4 and 5, Table 4).

**Table 4:** Flow reaction condition screening. Reaction conditions: (1 : 1) amine : *n*-BuLi, 27 °C, 0.83 M ester in dry THF, *R*_1_ and *R*_2_ = 0.075 mL

Entry	Ester	Stoichiometry amine : ester	*R*_t1_ (s)	*R*_t2_ (s)	Production time (min)	Conversion^a^ (%)
1	4c	1.0	10	5	4.1	64
2		1.5	10	6	3	99
3			20	12		83
4	4b	1.5	10	6	3	83
5			20	12		>99

a Conversions were calculated using ^1^H-NMR.

The transposition of Hevia's amide synthesis to flow clearly offers a technological advantage compared to the batch method, which requires air-sensitive techniques to avoid the risks associated with pyrophoric and highly reactive reagents. Flow setups, being closed systems by nature, drastically improve the safety of this method. Moreover, the ability to perform this reaction at room temperature within the same solvent system opens the door to larger scale processes, as costly cryogenic setups can be avoided. Motivated by these results, we explored the possibility of further increasing and intensifying the overall multi-step synthesis of mepivacaine (7).

### Continuous multi-step synthetic platform

The design of a continuous platform to synthesise mepivacaine was approached with a clear focus on sustainability, with aspects such as solvent, energy demand, waste generation and process safety considered to be critical parameters for scalability. Note that while the second step takes place in an organic solvent, the reductive amination is carried out in an aqueous environment, and an efficient solvent transfer needs to be implemented. While THF is clearly not ideal to extract the intermediate 4b from water, 2-MeTHF with its poor water solubility could be ideal to telescope the methylated ester into the amide coupling step.

Three key parameters were targeted for the optimisation of the first step: reactor temperature, concentration of the ester (4b) and residence time. Increasing the concentration of the ester to 100 mM (entry 1, Table 5) and even to 1.6 M (entry 4, Table 5), which had never been attempted before, showed that the system performs as efficiently as that at the 10 mM scale with exceptional potential for process intensification. In turn, decreasing the reactor temperature lowered the conversion (Table S9, ESI†); therefore, this was maintained at 150 °C. The system could still achieve full conversion even with significantly less equivalents of formic acid and formaldehyde (reduced to only 3.3 and 3.0, respectively), in line with what was previously reported.^22^ This not only improved the global sustainability of the process, but also enabled a steady operation of the next step (the amide coupling). Paraformaldehyde is an impurity present in the commercially available solution of 37% formaldehyde, which in increased amounts causes reactor fouling. However, reducing the equivalents of formaldehyde to 3.0 eq. prevents this from happening.

**Table 5:** Process intensification and condition optimisation of the reductive amination step. Reaction conditions: an appropriate concentration of methylpipecolinate (1b) + appropriate eq. of formaldehyde in H_2_O, and pH was adjusted to 4.0. Solution of formic acid in water. Reactions were performed at 8 bar and 150 °C. Conversions were calculated by ^1^H-NMR

Entry	[1b] (M)	Formaldehyde (eq.)	Formic acid (eq.)	Residence time (min)	Conversion (%)
1	0.10	27.9	2650 (neat)	45	>99
2	0.10	27.9	53	10	>99
3	0.83	16.2	6.4	5	>99
4	1.60	3.0	3.3	5	>99

Once the reductive amination was optimised, the goal was to couple it with the amide bond formation, ideally in a fully telescoped and continuous manner. A main concern arose: is the amide formation step robust enough to work in wet organic solvents? 4b was in fact efficiently extracted from the aqueous reaction environment in 2-MeTHF and could be used directly in the coupling step. This aspect is particularly important for scalability and cost efficiency; however, published protocols indicate that solvents are dried, distilled, and degassed, and indeed this was also used in our initial screening discussed above. However, by simply increasing the residence time from 12 seconds to less than 4 minutes and the *n*-BuLi equivalents from 1.5 to 2, conversions up to 90% under these suboptimal (but more practical) conditions were obtained (Table S13, ESI†).

**Fig. 3:**
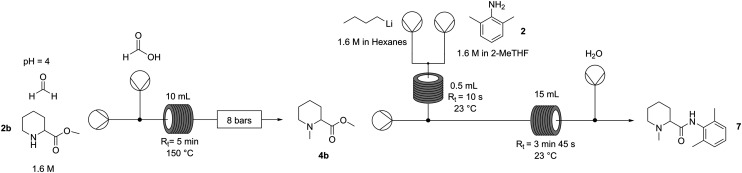
Overall continuous platform for the synthesis of mepivacaine. Process conditions: reductive amination – solution of methylpipecolinate (1b) 1.6 M in H_2_O at pH 4.0 + formaldehyde 3.0 eq. and formic acid 20% (v/v) 3.3 eq.; lithiation – solution of *n*-BuLi 1.6 M in hexanes 2 eq., solution of amine (2) 1.6 M in 2-MeTHF 1.0 eq; amide coupling – solution of *N*-methylpipecolinate (4b) 0.8 M in 2-MeTHF 1.0 eq., stream of Li-amide (5) 2.0 eq.

To prove the feasibility of the whole process (Fig. 3), a production campaign was run on a preparative scale: 7.2 grams (1.6 M) of pipecolic ester 1b were processed in 25 minutes with quantitative conversion and 93% crude yield of *N*-methylpipecolinate 4b. This was followed by a separate 16 min campaign to process 1.8 grams of the 4b intermediate, achieving in this step 90% conversion and 47% isolated yield of highly pure mepivacaine following a concomitant distillation–crystallisation procedure that allowed also the recovery of the unreacted amine as well as the 2-MeTHF used for the amide formation (Fig. 3). Given the 93% yield of the reductive amination, the platform afforded the anaesthetic (7) in 44% overall yield.

By following McElroy's recommendations to evaluate sustainability in the pharmaceutical industry,^35^ a detailed analysis of the individual steps and the whole process was performed (Table 6). Details of the calculations are given in the ESI.†

**Table 6:** Process efficiency metrics

Metric	Reductive amination	Lithiation	Amide coupling	Overall process
Space time yield (STY) (kg (L h)^−1^)	1.4	24.4	0.4	0.4
E factor (kg waste (kg product)^−1^)	13.5	1.1	4.0	18.6
Corrected E factor^a^ (kg waste (kg product)^−1^)	25.8	1.1	26.1	53.0
Process mass intensity (PMI) (kg total (kg product)^−1^)	26.8	2.1	27.1	56.0
Atom economy (%)	85	69	87	51

a Corrected E-factor includes the water contribution.

**Fig. 4:**
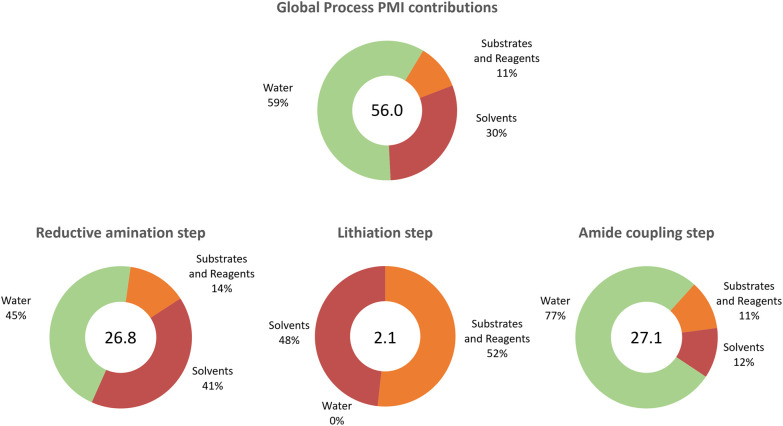
Process mass intensity details for the synthesis of mepivacaine (7).

We were delighted to see not only that the overall process was efficient in terms of cumulative space time yield (0.4 kg (L h)^−1^) but also that the environmental impact (E-factor) of the process was significantly lower (18.6 kg waste (kg product)^−1^) than those of the average pharmaceutical processes (25–100 kg waste (kg product)^−1^).^36^ In classical calculations of the E factor, the contribution of water is generally not included.^36^ Here, we also reported for comparison the corrected E factors (including water) and the difference in values is significant, with the obvious exclusion of the lithiation step. For the individual steps, the calculations show where the process reaches the highest efficiency in terms of productivity (lithiation step) and where there is a bottleneck (amide coupling). The E-factor showed again that the lithiation step is the most efficient step also in terms of waste management, highlighting the importance of the unit operation and solvent choice in the process design which is, in most cases, one of the biggest contributors to the process waste. Although the E-factor is a reliable measure of the environmental impact of a process, it focuses only on waste generation rather than resource management. Thus, including the process mass intensity (PMI) allows a deeper understanding of the resource consumption and identification of potential improvements rather than focusing only on problem solving.^37^ In Fig. 4, a detailed analysis of the PMI of the process is shown; in most cases, the water and the solvents used in the individual steps were the main contributors to the global index (56.0 kg total materials (kg product)^−1^). Considering that the average PMI values for commercial phase drugs range between 120 and 170,^38,39^ our process clearly demonstrates an improved efficiency and sustainability already at an early development stage of the synthetic methodology. While a direct comparison of this metric with that of previous methodologies (Fig. 1) is challenging, we attempted the same analysis which shows that this synthetic approach is beneficial (see Table S14†). This metric is a better tool that goes beyond waste minimisation to include efficient management of the resources,^40^ since it allows for individually targeting in further process optimisation rounds.

Additionally, for each step, the atom economy (AE) of the synthesis was calculated. The synthesis design is clearly highly efficient with two out of the three steps having more than 85% of the atoms of the reagents incorporated into the final product (7). When assessing the sustainability of the whole platform not only the above parameters were considered but also the solvent choice. Here, 2-MeTHF, a bio-derived ether,^41^ replaced tetrahydrofuran (THF), a classical solvent used in organometallic chemistry. This choice could potentially prevent future fossil fuel shortages.

## Conclusions

The 2020 COVID-19 pandemic exposed the fragility of global supply chains including in the chemical sector. Hospitals faced shortages of local anaesthetics like mepivacaine during the pandemic. This work presents an alternative and quickly implementable method for synthesising this API.

This multi-step continuous platform has proved to be more sustainable than the previously reported methods. Notably, the use of water as the exclusive solvent for the reductive amination step and formic acid as a reducing agent has eliminated the need for atom-inefficient reagents like 2-picoline-borane or the potentially hazardous sodium cyanoborohydride.

In the case of the amide coupling, the main advantage of using flow chemistry lies in the ability to re-open the chemical window of lithiation at room temperature, thanks to the excellent heat transfer in the tubular reactor. From a process chemistry perspective, the use of flow chemistry not only allows the reaction to occur at room temperature but also provides efficient control over the reaction exothermicity, thereby avoiding potential runaway scenarios on large scales. Even though a significant amount of material is involved, the effective volume engaged at any given time in the reactor is minimal compared to what would be required in batch mode.

The role of 2-MeTHF is not limited to making the synthesis more sustainable and avoiding intermediate solvent exchanges; it also plays a crucial role in the feasibility of the process, minimising reactor fouling. 2-MeTHF not only readily extracts the *N*-methylated ester from the aqueous reaction environment, but it can also solubilise LiOH which is known to cause clogging issues when handling organometallics in flow.

While we have reported here a telescoped approach between the first and second step of this cascade, the extraction of *N*-methylpipecolinate is carried out manually due to the limitations of our flow system (Scheme S6†), and this could be clearly done in an automated manner with more advanced equipment.

Our current work focuses on expanding this methodology to the whole range of this class of anaesthetics and on the automation of the unit operation. Additionally, efforts are being made to explore large scale implementation of the process.

To summarise, we have demonstrated a new and highly sustainable synthetic platform for the synthesis of the local anaesthetic mepivacaine (7). Given the modularity of the flow setup, this could be extended to the synthesis of the rest of the caine family such as bupivacaine, lidocaine, ropivacaine, *etc*., which also contain the amide core in their structures,^14,42,43^ demonstrating in this way the wide application range of our method.

## Experimental

### General

Flow reactions were carried out using Vapourtec E- and R-series. ^1^H and ^13^C NMR were recorded in 300 and 400 MHz instruments. High resolution mass spectrometry experiments were conducted in positive mode with determination of elemental composition by nanoelectrospray-MS or EI-MS analysis on Orbitrap. Reagents and solvents were obtained from commercial suppliers and used without additional purification.

### Experimental procedure for batch tests

#### Reductive amination

A solution of pipecoline derivative (1c) or (1b) in water was mixed with a specific amount of formaldehyde and the pH was adjusted to 4.0 with 15% (v/v) acetic acid. Then, a specific amount of the reducing agent was added, the temperature was adjusted, and the reaction was monitored by ^1^H-NMR.

#### Amide coupling

Solvents used were previously distilled and degassed. All the reactions were performed using inert atmosphere techniques. Step 1: Lithium amide formation. To a flame dried Schlenk tube, 5.6 mL of hexane and 2,6-dimethylaniline (2) (3 mmol) were added under magnetic stirring. Finally, *n*-BuLi (3 mmol) was introduced; instantaneously a white yellow suspension appeared. The mixture was left undisturbed for one hour. Then, the solvent was removed, and the Li-amide (5) was obtained as a white yellow solid. Step 2: To the previously obtained solid, 3.0 mL of 2-MeTHF were added to obtain a 1.0 M solution. To a Schlenk tube previously flame-dried and put under argon, appropriate amounts of pipecolic ester (4b) and 2-MeTHF were added. Then, the required amount of the previously prepared Li-amide (5) solution was introduced and left under vigorous stirring for a specific amount of time. The reaction was quenched by addition of a saturated solution of NH_4_Cl (3.0 mL), and the product was extracted with 2-MeTHF and evaporated *in vacuo* to afford the final coupling product. Conversions were calculated by ^1^H-NMR.

Remarks: when the solvent used for the deprotonation and the coupling step is 2-MeTHF, the intermediate solvent removal is not performed.

### Experimental procedures for flow reactions

#### Reductive amination

A solution of the desired concentration of the pipecolic derivative and appropriate equivalents of formaldehyde was prepared in water and the pH was adjusted to 4.0 using either 2 M NaOH or acetic acid. A solution of formic acid was prepared using water as the sole solvent. Both solutions were then pumped into a tubular reactor at the flow rates and temperature of each specific assay. Reactions were performed at 8 bar and conversions were calculated by ^1^H-NMR. For the assays where the final yield is reported, the reaction crude was basified using either NaOH or NaHCO_3 (s)_ to pH 10 and extracted with 2-MeTHF, and the solvent was removed by rotatory evaporation.

Remarks: it is important to use fresh bottles of 37% formaldehyde to avoid substantial amounts of paraformaldehyde that may lead to clogging issues downstream.

#### Lithiation and amide coupling

The solutions of ester and 2,6-dimethylaniline were prepared either using dried and distilled solvents or with commercial solvents previously passed through molecular sieves, degassed and kept under an inert atmosphere. The solution of *n*-BuLi was used as obtained from the supplier. The solutions of *n*-BuLi and amine were then introduced into the first reactor and then continuously mixed with the ester stream at the flow rates of each specific assay and at RT (23 °C). Conversions were calculated using ^1^H-NMR. When final yields were reported, the biphasic mixture was introduced without further modification in a round-bottom flask and distilled under atmospheric pressure; when the different fractions were pulled, the distilling flask was allowed to cool down to room temperature and the crystals were collected and washed with cold water 3 × 5 mL.

Remarks: it is crucial to use fresh bottles of *n*-BuLi since they tend to have lower amounts of LiH which can potentially lead to reactor fouling. We also found that bottles from Across were cleaner in terms of solid particles than the ones from Sigma Aldrich.

### Overall flow setup for the synthesis of mepivacaine (7)

#### Reductive amination

A solution containing methylpipecolinate (2b) (1.6 M) and 3.0 eq. of formaldehyde was prepared in water and the pH was adjusted to 4.0 using 2 M NaOH. A 20% (v/v) formic acid solution was prepared using water as the sole solvent. Each solution was then pumped into a heated (10 mL, 150 °C, 5 min) tubular reactor at a flow rate of 1 mL min^−1^ to achieve a residence time of 5 min; to keep the system pressurised at 8 bar, a back-pressure regulator was placed downstream. After reaching the steady state (3× *R*_t_ = 15 min), the product was collected for 25 min, then basified with 2 M NaOH (30 mL) to pH 10, extracted 3 × 25 mL with 2-MeTHF, and dried with Na_2_SO_4_ (2 g); the solvent was removed by rotatory evaporation to obtain the crude product (4b) in 93% yield.

#### Lithiation and amide coupling

The following solutions were prepared: 0.8 M solution containing the previously synthetized ester (4b) (without any purification) and 1.6 M 2,6-dimethylaniline (2), both in technical-grade 2-MeTHF previously degassed and passed over 4 Å molecular sieves; the solutions were kept under an Ar atmosphere. The solution of *n*-BuLi (1.6 M) was used as obtained from the supplier. Running the reaction: the solutions of *n*-BuLi (2 mL min^−1^) and amine (2) (1 mL min^−1^) were then introduced into the first tubular reactor (0.5 mL, 23 °C, 10 s), then continuously mixed with a stream of ester (4b) (1 mL min^−1^) in the second tubular reactor (15 mL, 23 °C, 3 min 45 s), and the last inlet with a water stream (1 mL min^−1^) used for quenching the excess *n*-BuLi and Li-amide was placed downstream. After reaching the steady state (3× *R*_t_ = 11 min 15 s), the crude product (7) was collected for 16 min. Then, the biphasic mixture was introduced in a round-bottom flask without further modification and distilled under atmospheric pressure; once the different fractions were pulled, the distilling flask was allowed to cool down to room temperature and the crystals were collected and washed with cold water (3 × 5 mL), to finally afford the final product (7) in 47% yield.

## Author contributions

FP conceptualised the work, secured funding, and supervised the project. PDK established the methodology, carried out all the experiments and analysed the data. PDK wrote the original draft. FP and PDK reviewed the manuscript for submission.

## Conflicts of interest

There are no conflicts to declare.

## Supplementary Material

GC-026-D3GC04375D-s001

GC-026-D3GC04375D-s002
