# Supplementary material for: Rapid production of the anaesthetic mepivacaine through continuous, portable technology
Source: Green Chem. 2024 Jan 23;26(4):2313–21. doi: 10.1039/d3gc04375d (PMC10875724; doi:10.1039/d3gc04375d)
Supplement: GC-026-D3GC04375D-s002 [file GC-026-D3GC04375D-s002.pdf]

Electronic Supplementary Material (ESI) for Green Chemistry.
This journal is © The Royal Society of Chemistry 2024

# Electronic Supporting information

## Rapid production of the anaesthetic mepivacaine through continuous, portable technology

Pablo Diaz-Kruik [a] and Francesca Paradisi,*[a]
[a]Department of Chemistry Biochemistry and Pharmaceutical Sciences, University of Bern, Switzerland.

Table of Contents

**Reductive amination screening flow set-up** 2
Pressure effect 2
Formic acid equivalents 2
Temperature and residence time effect 2
**Amide bond formation through acyl fluoride intermediate (batch)** 3
**Amide bond formation biocatalytic attempt (batch)** 3
**Process intensification and optimization of the reductive amination (flow)** 4
Conditions screening at 0.10 M of methylpipecolinate (1b) (Residence time effect) 4
Conditions screening at 0.83 M of methylpipecolinate (1b) 4
Conditions screening at 1.6 M of methylpipecolinate (1b) 4
**Amide Bond formation through Li-amide formation (Flow)** 5
Electrophilic quench semi-continuous mode 5
**Telescoped amide bond formation reaction screening:** 5
Residence time effect 5
n-BuLi and Li-amide ratio effect 6
Fully telescoped screening 6
Fully continuous set-up 6
**Green metrics** 6
Equation S1 Space time yield (STY) 6
Equation S2 E factor 6
Equation S3 Process mass intensity (PMI) 6
Equation S4 Mass intensity (MI) 7
Equation S5 Atom economy (AE) 7
Comparison between previously existing methods and this work 7
Additional considerations for green metrics calculations 7
**Structural Characterization of *N*-methylpipecolinate (4b)** 8
$^{1}$H-NMR (4b) 8
$^{13}$C-NMR (4b) 9
High resolution mass spectrometry (4b) 9
**Structural Characterization of mepivacaine (7)** 10
$^{1}$H-NMR (7) 10

$^{13}$C-NMR (7) 11

High resolution mass spectrometry (7) 11

**References** 12

## Reductive amination screening flow set-up

To find the optimal conditions for the continuous reductive amination the following parameters were targeted: pressure, formic acid equivalents, temperature, and residence time.

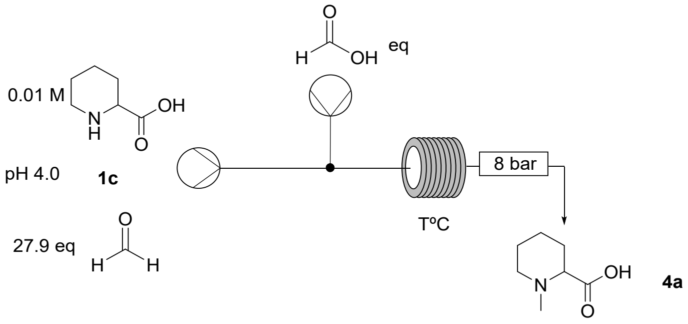


Scheme S1 Flow set-up for the reductive amination step

### Pressure effect

| Entry | Residence Time (min) | Formaldehyde (eq.) | Molar Ratio (Pipecolic acid: Formic acid) eq. | Temperature (ºC) | BPR (8 bars) | Conversion (%)* |
|---|---|---|---|---|---|---|
| 1 | 45 | 27.9 | 25 | 90 | No | nd |
| 2 | | | | | Yes | nd |
| 3 | | | | 120 | No | nd |
| 4 | | | | | Yes | 3 |

Table S1 Conditions: Pipecolic acid (1c) 10 mM, formic acid 250 mM. *Conversion calculated by $^{1}$H-NMR

### Formic acid equivalents

| Entry | Residence Time (min) | Formaldehyde (eq.) | Molar Ratio (Pipecolic acid: Formic acid) eq. | Temperature (ºC) | BPR (bars) | Conversion (%)* |
|---|---|---|---|---|---|---|
| 1 | 45 | 27.9 | 55 | 120 | Yes | 4 |
| 2 | | | 2650 (Neat) | | | 33 |

Table S2 Conditions: Pipecolic acid (1c) 10 mM, formic acid 250 mM. *Conversion calculated by $^{1}$H-NMR

### Temperature and residence time effect

| Entry | Residence Time (min) | Formaldehyde (eq.) | Molar Ratio (Pipecolic acid: Formic acid) eq. | Temperature (ºC) | BPR (bars) | Conversion (%)* |
|---|---|---|---|---|---|---|
| 1 | 45 | 27.9 | 2650 (Neat) | 150 | 8 | >99 |

| | | | | | | |
|---|---|---|---|---|---|---|
| 2 | 15 | 27.9 | 2650 (Neat) | 150 | 8 | 80 |

Table S3 Conditions: Pipecolic acid (1c) 10 mM. *Conversion calculated by $^{1}$H-NMR

Formic acid equivalents vs residence time

| Entry | Residence Time (min) | Formaldehyde (eq.) | Molar Ratio (Pipecolic acid: Formic acid) eq. | Temperature (ºC) | BPR (bars) | Conversion (%)* |
|---|---|---|---|---|---|---|
| 1 | 45 | 27.9 | 1325 (50% in H2O) | 150 | 8 | >99 |
| 2 | 15 | 27.9 | 1325 (50% in H2O) | 150 | 8 | 61 |
| 3 | 45 | 27.9 | 265 (10% in H2O) | 150 | 8 | 84 |
| 4 | 15 | 27.9 | 265 (10% in H2O) | 150 | 8 | 62 |

Table S4 Conditions: Pipecolic acid (1c) 10 mM. *Conversion calculated by $^{1}$H-NMR.

## Amide bond formation through acyl fluoride intermediate (batch)

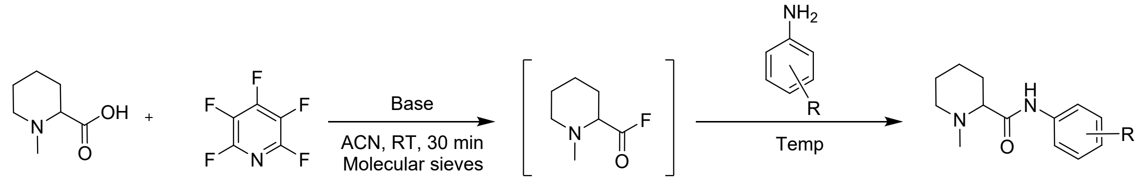


1c PFP Not Isolated

Scheme S2 Amide bond formation trough acyl fluoride intermediate

| Entry | PFP (eq.) | Base (eq.) | Amine (eq.) | Activation time (min) | Total reaction time | Conversion* (%) |
|---|---|---|---|---|---|---|
| 1 | 1.1 | DIPEA (2) | O-Toluidine (1.0) | 30 | 1.5 days | 33 |
| 2 | 1.1 | DIPEA (4) | O-Toluidine (1.0) | 30 | 4 days | 31 |
| 3 | 1.1 | DBU (2) | O-Toluidine (1.0) | 30 | 4 days | 22 |
| 4 | 1.1 | DIPEA (2) | O-Toluidine (1.5) | 30 | 4 days | 44 |
| 5 | 3.0 | DIPEA (2) | O-Toluidine (1.0) | 30 | 4 days | 73 |

Table S5 Reaction conditions: 250 mM (1c), 25 °C. *Conversion measured by HPLC

## Amide bond formation biocatalytic attempt (batch)

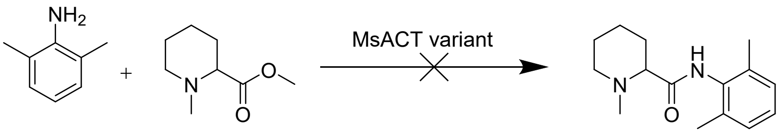


2 4b 7

Scheme S3 Biocatalytic amine bond formation using Mycobacterium Smegmatis (MsACT)

| Entry | Acyl donor | Substrate | Enzyme | Conversion (%)* |
|---|---|---|---|---|

| | | | | |
|---|---|---|---|---|
| 1 | (**4b**) (10 mM) | 2,6-Dimethylaniline (250 mM) (25 eq.) | MsAcT (WT) | nd |
| 2 | | | MsAcT-S11C | nd |

Table S6 Conditions : [Enzyme] = 2.75 mg/mL, phosphate buffer 100 mM pH 8.0, 45 ºC, 48h. *Conversions calculated by HPLC. Enzymes were obtained following the protocols from the previous literature.[1]

## Process intensification and optimization of the reductive amination (flow)

### Conditions screening at 0.10 M of methylpipecolinate (1b) (Residence time effect)

| Entry | Residence Time (min) | Formaldehyde (eq.) | Molar Ratio (methylpipecolinate: Formic acid) eq. | Temperature (ºC) | BPR (bars) | Conversion (%)* |
|---|---|---|---|---|---|---|
| 1 | 45 | 27.9 | 53 | 150 | 8 | >99 |
| 2 | 20 | 27.9 | 53 | 150 | 8 | >99 |
| 3 | 10 | 27.9 | 53 | 150 | 8 | >99 |
| 4 | 1 | 27.9 | 53 | 150 | 8 | 39 |

Table S7 Reaction conditions: 0.1 M methylpipecolinate (1b) + 27.9 eq. of formaldehyde (pH adjusted with 15 % v/v solution of acetic acid). 20 % (v/v) solution of formic acid in water. Reactor volume was 10 mL for entries 1-3 and 1.2 mL for entry 4. Reactions were performed at 8 bars. Conversion were calculated by $^1$H-NMR.

### Conditions screening at 0.83 M of methylpipecolinate (1b)

| Entry | Residence Time (min) | Formaldehyde (eq.) | Molar Ratio (Methylpipecolinate Formic acid) eq. | Temperature (ºC) | BPR (bars) | Conversion (%)* |
|---|---|---|---|---|---|---|
| 1 | 5 | 16.2 | 6.4 | 150 | 8 | > 99 |
| 2 | 10 | 16.2 | 6.4 | 150 | 8 | > 99 |

Table S8 Reaction conditions: 0.83 M methylpipecolinate (1b) + 16.2 eq. of formaldehyde (pH adjusted with 1 M solution of sodium acetate). 20 % (v/v) solution of formic acid in water. Reactor volume 1.16 mL . Reactions were performed at 8 bars. Conversions were calculated by $^1$H-NMR.

### Conditions screening at 1.6 M of methylpipecolinate (1b)

| Entry | Residence Time (min) | Formaldehyde (eq.) | Molar Ratio (Methylpipecolinate: Formic acid) eq. | Temperature (ºC) | BPR (bars) | Conversion (%)* |
|---|---|---|---|---|---|---|
| 1 | 5 | 8.4 | 6.4 | 40 | 8 | n.d. |
| 2 | 5 | 8.4 | 6.4 | 100 | 8 | 28 |
| 3 | 5 | 8.4 | 6.4 | 150 | 8 | >99 |
| 4 | 5 | 3 | 3.3 | 150 | 8 | >99 |

Table S9 Reaction conditions: 1.6 M methylpipecolinate (1b) + 8.4 eq. of formaldehyde (pH adjusted with 1 M solution of sodium acetate). 20 % (v/v) solution of formic acid in water. Reactor volume 1.5 mL . Reactions were performed at 8 bars. Conversions were calculated by $^1$H-NMR.

## Amide Bond formation through Li-amide formation (Flow)

Electrophilic quench semi-continuous mode

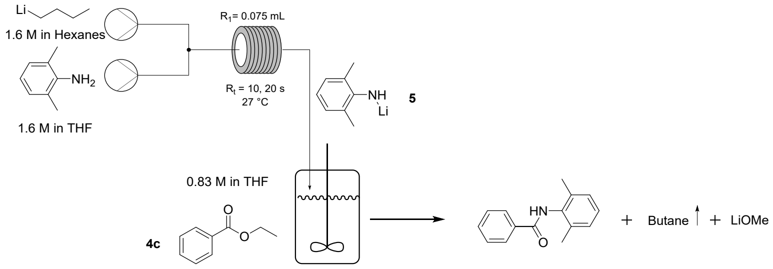


Scheme S4 Semi-continuous set-up for the amide coupling reaction.

| Entry | Stoichiometry (Amine: n-BuLi) | Residence time (sec) | Collection time in ``STR´´ (min) | Volume of electrophile (mL) | Temperature (°C) | Conversion* (%) |
|---|---|---|---|---|---|---|
| 1 | | 1 | | 20 | 27 °C | 57 |
| 2 | 1:1 | 10 | 2 | 2 | 27 °C | >99 |
| 3 | | 20 | | 2 | 27 °C | >99 |

Table S10. Conditions: 0.075 mL of reactor volume, [2,6-dimethylaniline] = 2.5 M, [n-BuLi] = 2.5 M, [Ethyl benzoate] = 0.83 M, dry and degassed THF was used, quench was performed with 1 mL of $H_2O$ straight after collection was completed. *Conversions were calculated by $^1$H-NMR

## Telescoped amide bond formation reaction screening:

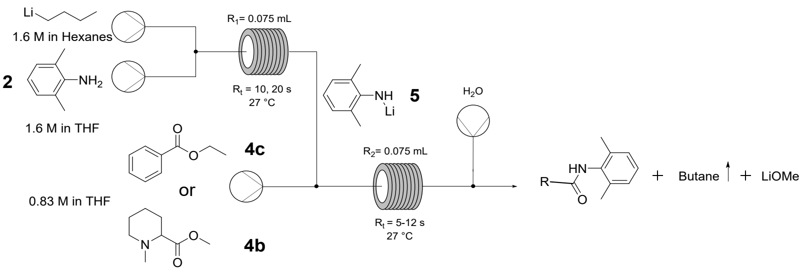


Scheme S5 Flow set-up for the amide formation

Residence time effect

| Entry | Stoech (n-Buli: amine) | Stoech (Li-amide: N-methylpipecolinate) | R1 (sec) | R2 (sec) | Conversion (%) |
|---|---|---|---|---|---|
| 1 | | | 1 | 0.6 | 26 |
| 2 | 1:1 | 1.5:1 | 5 | 3 | 37 |
| 3 | | | 10 | 7 | 52 |
| 4 | | | 20 | 15 | 54 |

Table S11 Reaction conditions: N-methylpipecolinate (4b) (0.8 M), n-Buli (1.6 M), 2.6-dimethylaniline (1.6 M), reactor volumes 0.075 mL, reaction temperature 23 °C. Conversions were calculated by $^1$H-NMR

n-BuLi and Li-amide ratio effect

<table>
<tr><th>Entry</th><th>Stoech (n-Buli: amine)</th><th>Stoech (Li-amide: N-methylpipecolinate)</th><th>R1 (sec)</th><th>R2 (sec)</th><th>Conversion (%)</th></tr>
<tr><td>1</td><td rowspan="2">1.5:1</td><td>2:1</td><td rowspan="4">10</td><td>7</td><td>57</td></tr>
<tr><td>2</td><td>1.5:1</td><td>6</td><td>51</td></tr>
<tr><td>3</td><td rowspan="2">2:1</td><td>2:1</td><td>7</td><td>91</td></tr>
<tr><td>4</td><td>5:1</td><td>8</td><td>60</td></tr>
</table>

Table S12 Reaction conditions: N-methylpipecolinate (4b) (0.8 M), n-Buli (1.6 M), 2.6-dimethylaniline (1.6 M), reactor volumes 0.075 mL, reaction temperature 23 °C. Conversions were calculated by $^1$H-NMR

Fully telescoped screening

<table>
<tr><th>Entry</th><th>Stoech (n-Buli: amine)</th><th>Stoech (Li-amide: N-methylpipecolinate)</th><th>R1 (sec)</th><th>R2 (sec)</th><th>Conversion (%)</th></tr>
<tr><td>1</td><td rowspan="2">2:1</td><td rowspan="2">2:1</td><td rowspan="2">10</td><td>7</td><td>42</td></tr>
<tr><td>2</td><td>225</td><td>90</td></tr>
</table>

Table S13 Solution of n-BuLi 1.6 M in Hexanes, solution of amine (**2**) 1.6 M in 2-MeTHF. **Amide coupling.** Solution of N-methylpipecolinate (**4b**) 0.8 M in 2-MeTHF. 2-MeTHF was used without additional purification.

Fully continuous set-up

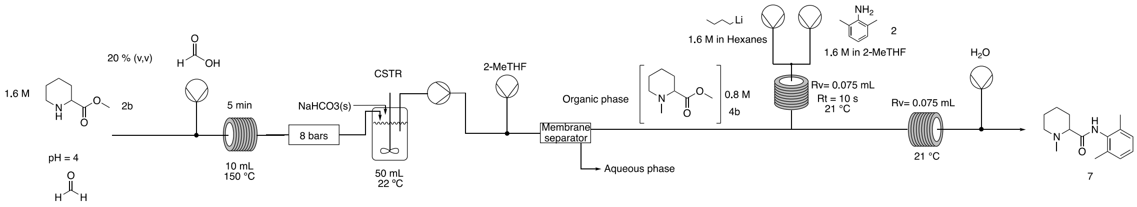


*Scheme S6 Fully continuous set-up* for the synthesis of mepivacaine. Process conditions: **Reductive amination.** Solution of methylpipecolinate **(1b)** 1.6 M in $H_2O$ pH 4.0 + Formaldehyde 3.0 eq. Formic acid 20 % (v,v) 3.3 eq. **Lithiation**. Solution of n-BuLi 1.6 M in Hexanes 2eq., solution of amine (**2**) 1.6 M in 2-MeTHF 1.0 eq. **Amide coupling.** Solution of N-methylpipecolinate (**4b**) 0.8 M in 2-MeTHF 1.0 eq., stream of Li-amide (**5**) 2.0 eq.

Full coupling of the overall system in continuous was optimal since the gas (butane) generated in the lithiation step caused an intermittent back flow of the organic phase into the membrane separator leading to a very unstable and challenging set-up. Nonetheless, conversions reached ~45%. The placement of a check valve downstream of the membrane separator did not improve the result.

## Green metrics

All the calculations have been performed following the recommendations and assumptions from McElroy et al., 2015.[2] and others.[3,4]

Equation S1 Space time yield (STY)

$$Space - time\ yield\ (STY) = \frac{Mass\ of\ product\ (Kg)}{Reaction\ time\ (h) \cdot Reactor\ volume\ (L)}$$

Equation S2 E factor

$$E\ factor = \frac{Total\ waste\ (Kg)}{Total\ product\ (Kg)}$$

Equation S3 Process mass intensity (PMI)

$$Process\ mass\ intensity\ (PMI) = \frac{Total\ raw\ materials\ used\ in\ process\ (Kg)}{Total\ product\ (Kg)} = \sum [MI]_{solvents,\ reagents, water, etc}$$

Equation S4 Mass intensity (MI)

$$Mass\ intensity\ (MI) = \frac{Raw\ material\ used\ (Kg)}{Total\ product\ \ (Kg)}$$

Equation S5 Atom economy (AE)

$$Atom\ economy\ (AE) = \frac{Molecular\ weight_{product}}{\sum Molecular\ weigth_{Starting\ materials}} \cdot 100$$

Comparison between previously existing methods and this work

| Metric | This work | Ekenstam *et al.*, 1957 | Suveges *et al.*, 2017 |
|---|---|---|---|
| Space time yield (STY) kg·(L·h)$^{-1}$ | 0.4 | 0.037 | 0.077 |
| E factor (kg waste·(Kg product)$^{-1}$) | 18.6 | 7.5 | 496.7 |
| Corrected E factor (kg waste·(Kg product)$^{-1}$)* | 53.0 | 36.2 | 719.2 |
| Process mass intensity (PMI) (Kg total·(Kg product)$^{-1}$) | 56.0 | 39.2 | 721.2 |

Table S14 overall metrics for the different analyzed processes. For a more detailed breakdown of each step refer to supplementary excel file. Note that the lower E factors and PMI's reported for the Ekenstam method are not 100% accurate since crucial data was missing for workup and purification stages. Presumably the values would significantly increase. *Corrected E factor includes the water contribution.

Remarks:

- For PMI calculations on *Suveges et al.,* 2017 method, extraction and base volumes for last step are not reported but, taking into account the final volume of the solution, we assume (at least) the same volumes as for their 1s step.
- For Ekenstam *et al., 1957*: platinum oxide not considered since it is recovered.
- Wash volumes not known same for recrystallization.
- For clarity the nomenclature of each molecule is kept as in the original manuscripts.

Additional considerations for green metrics calculations

- Work-up, isolation and purification steps are included in the calculations.

**Space-Time-Yield (STY)**

- STY calculated considering isolated yields (whenever the intermediate/product was isolated)
- For the global STY the lower STY value of the whole process has been taken since it is the bottleneck of the productivity.

**Process Mass Intensity (PMI)**

The following considerations and assumptions were used:

- 25 minutes of production (reductive amination)
- 16 minutes of production for (Amide coupling and Lithiation)
- Total amount of water ``**in**´´ refers to the total amount of water used in each single step.
- Up to 90% of the used solvents can be recycled.
- For the lithiation step the solvent and 2,6-dimethylaniline are fully telescoped into the next step and both are recycled.

**E-factor**

- Corrected E factor includes the water contribution.

## Structural Characterization of *N*-methylpipecolinate (4b)

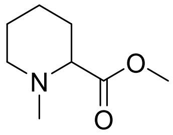

**4b**

$^{1}$H-NMR (4b)

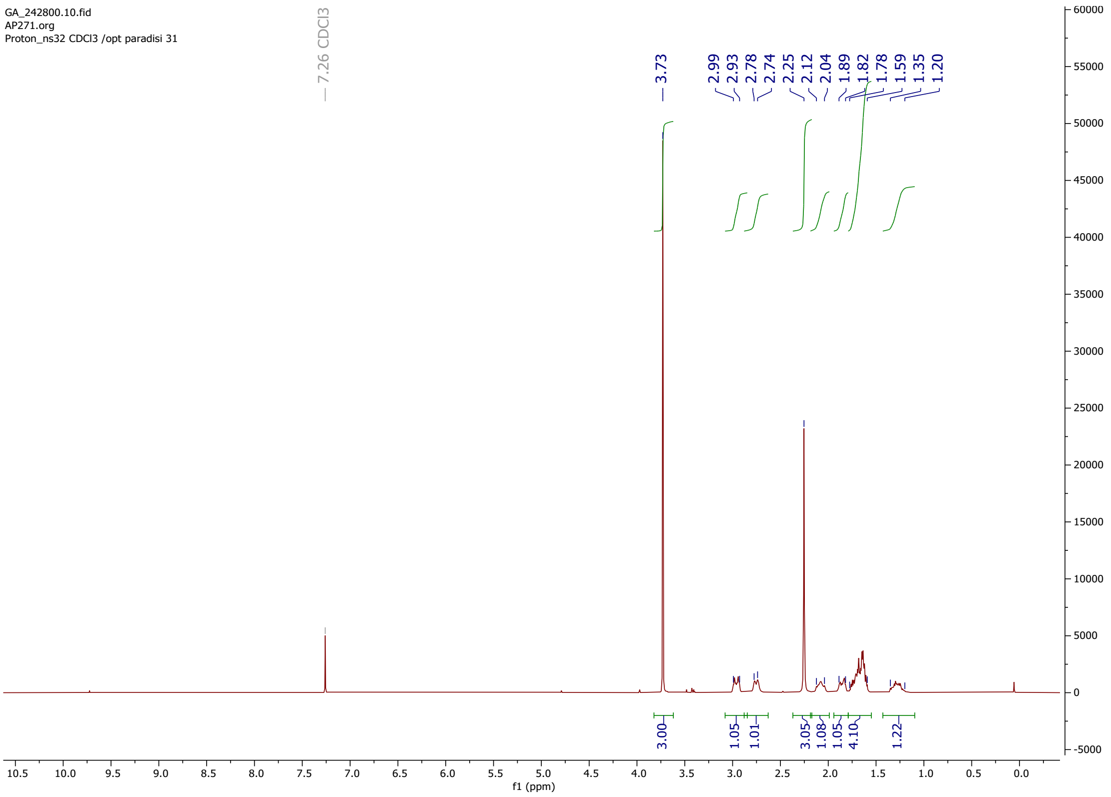


$\delta_H$ (300 MHz, $CDCl_3$) 3.73 (3 H, s), 2.96 (1 H, d, *J* 19.3), 2.76 (1 H, d, *J* 10.9), 2.25 (3 H, s), 2.08 (1 H, d, *J* 25.2), 1.85 (1 H, d, *J* 20.5), 1.68 (4 H, d, *J* 55.3), 1.27 (1 H, d, *J* 45.3).

$^{13}$C-NMR (4b)

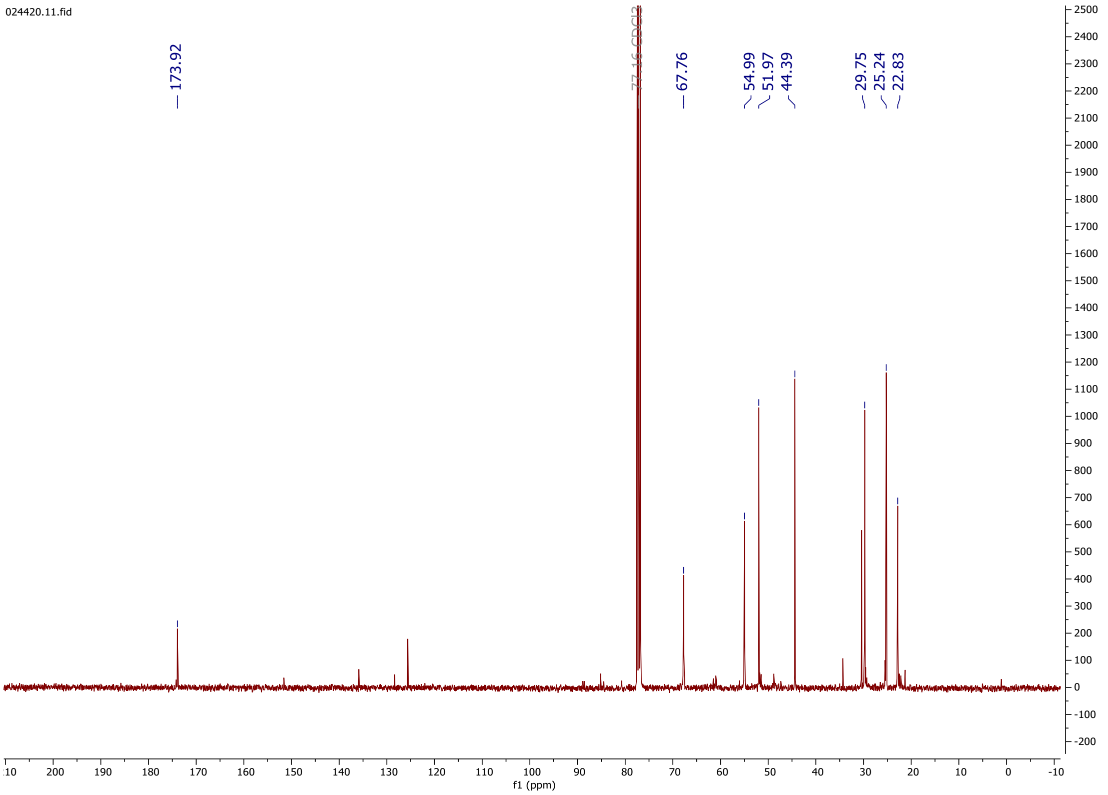


$^{13}$C NMR (101 MHz, $CDCl_3$) δ 173.92, 67.76, 54.99, 51.97, 44.39, 29.75, 25.24, 22.83.

High resolution mass spectrometry (4b)

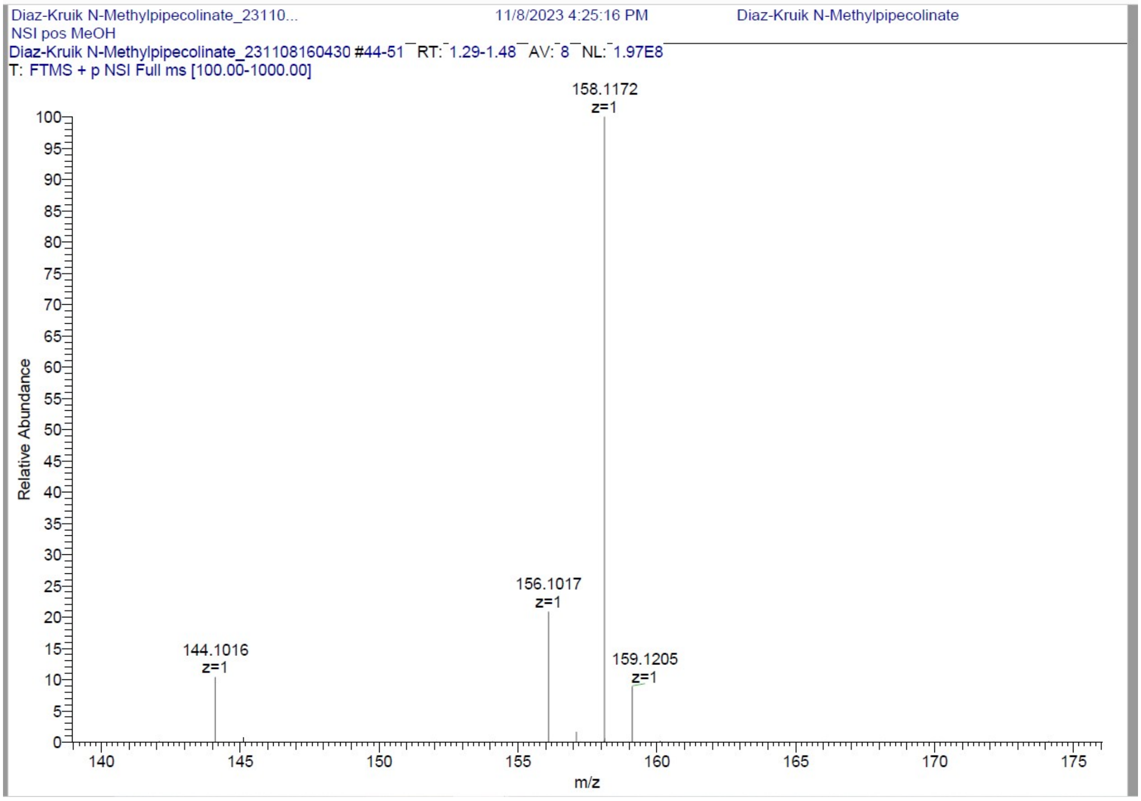

Elemental composition search on mass 158.1172

m/z= 153.1172-163.1172

| m/z | Theo. Mass | Delta (ppm) | Composition |
|---|---|---|---|
| 158.1172 | 158.1176 | -2.18 | $C_8H_{16}O_2N$ |

Figure S1 mass spectrometry (4b)

*m/z* 158.1172 ($M^+$ + H, 100%)

Delta ppm: 2.18

Type of analysis: +ESI-MS

## Structural Characterization of mepivacaine (7)

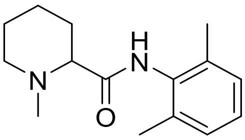

**7**

$^{1}$H-NMR (7)

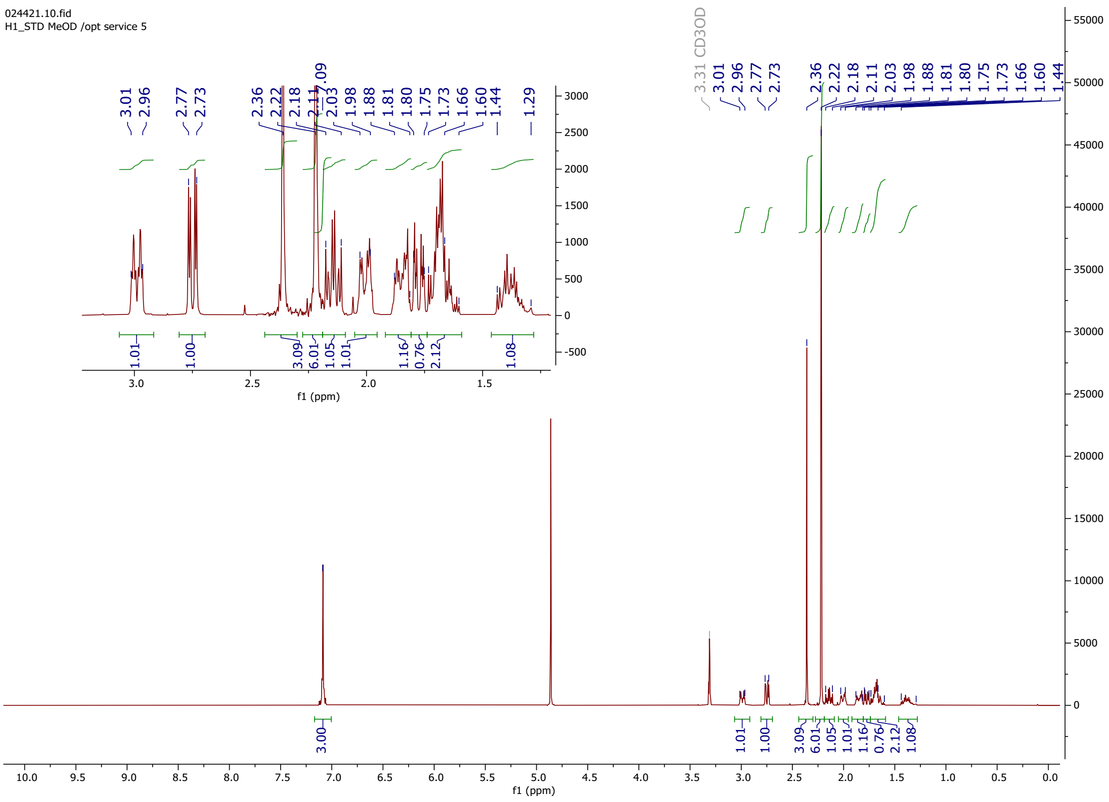


δ $_H$ (400 MHz, MeOD) 7.17 – 7.00 (3 H, m), 2.99 (1 H, dt, *J* 11.6, 3.6), 2.75 (1 H, dd, *J* 11.0, 3.1), 2.36 (3 H, s), 2.22 (6 H, s), 2.19 – 2.09 (1 H, m), 2.05 – 1.95 (1 H, m), 1.92 – 1.81 (1 H, m), 1.77 (1 H, m), 1.74 – 1.59 (2 H, m), 1.46 – 1.28 (1 H, m).

$^{13}$C-NMR (7)

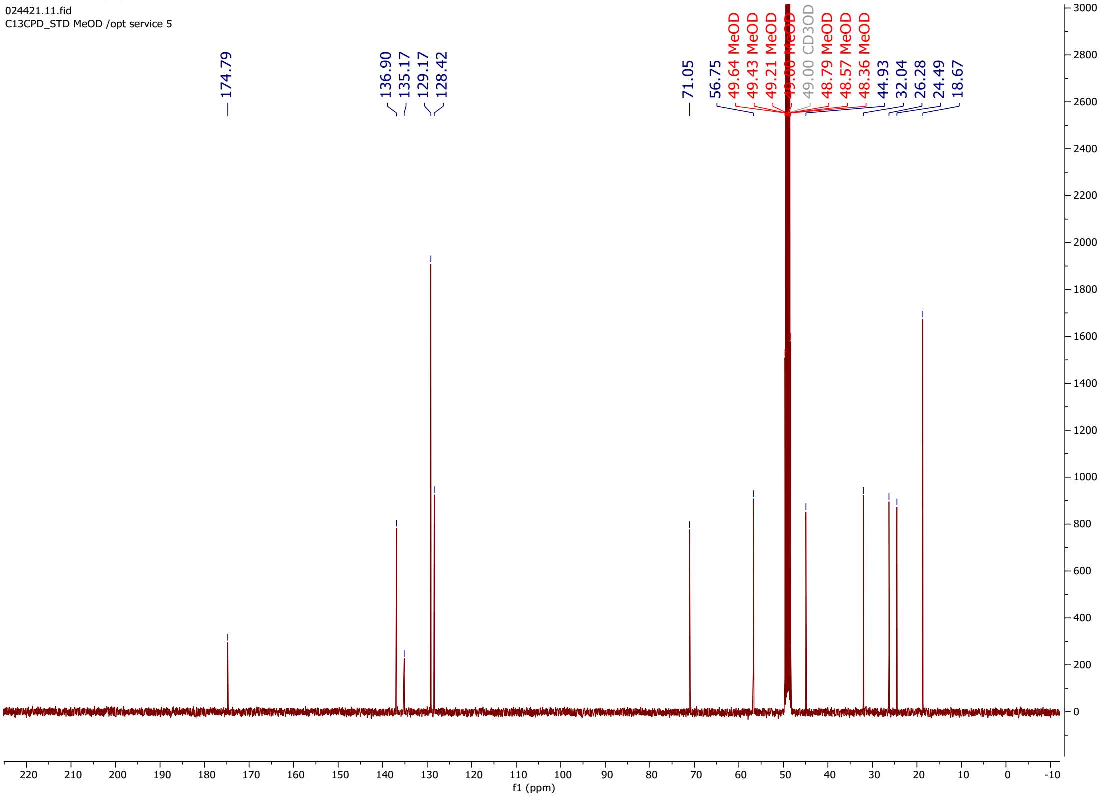


$^{13}$C NMR (101 MHz, MeOD) δ 174.79, 136.90, 135.17, 129.17, 128.42, 71.05, 56.75, 44.93, 32.04, 26.28, 24.49, 18.67.

High resolution mass spectrometry (7)

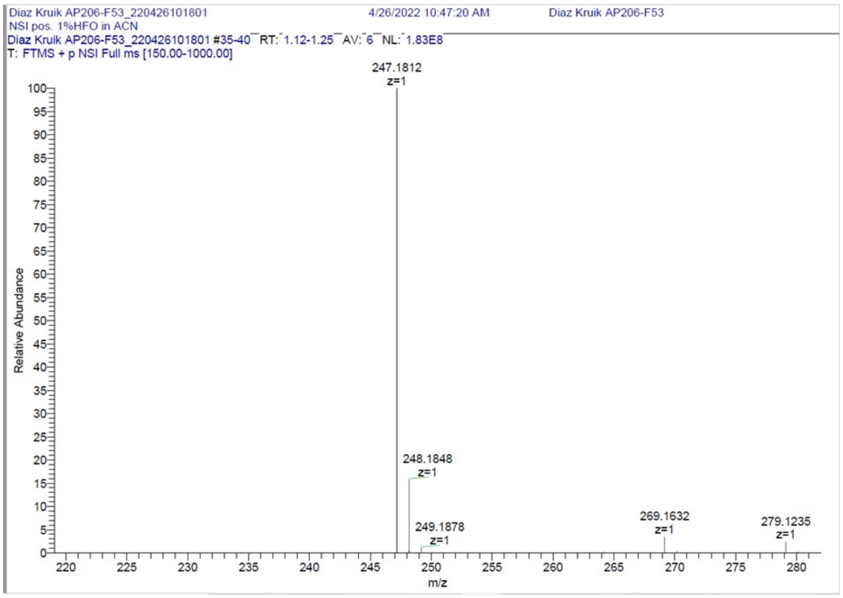


Elemental composition search on mass 247.1812

m/z= 242.1812-252.1812

| m/z | Theo. Mass | Delta (ppm) | Composition |
|---|---|---|---|
| 247.1812 | 247.1805 | 3.03 | $C_{15}H_{23}ON_2$ |
| | 247.1781 | 12.77 | $C_{13}H_{24}ON_2Na$ |
| | 247.1778 | 13.88 | $C_{12}H_{25}O_4N$ |

Figure S2 mass spectrometry (7)

*m/z* 247.1812 ($M^+$ + H, 100%)

Delta ppm: 3.03

Type of analysis: +ESI-MS

## References


1 M. L. Contente, D. R. Padrosa, F. Molinari and F. Paradisi, *Nat. Catal.*, 2020, **3**, 1020–1026.

2 C. R. McElroy, A. Constantinou, L. C. Jones, L. Summerton and J. H. Clark, *Green Chem.*, 2015, **17**, 3111–3121.

3 C. H. Benison and P. R. Payne, *Curr. Res. Green Sustain. Chem.*, 2022, **5**, 100229.

4 A. Lapkin and D. J. C. Constable, *Green Chemistry Metrics: Measuring and Monitoring Sustainable Processes*, 2009.
